# Parent–Child Communication About Potentially Traumatic Events: A Systematic Review

**DOI:** 10.1177/15248380231207906

**Published:** 2023-11-09

**Authors:** Mèlanie Sloover, Sabine E. M.J. Stoltz, Elisa van Ee

**Affiliations:** 1 Radboud University Nijmegen, The Netherlands; 2 Psychotraumacentrum Zuid Nederland, Reinier van Arkel, s-Hertogenbosch, The Netherlands

**Keywords:** PTSD, support seeking, sexual assault, attachment, reporting/disclosure, treatment/intervention, child abuse, cultural contexts

## Abstract

Social support plays an important role in children’s well-being after experiencing a potentially traumatic event (PTE). One such source of support is the parent–child relationship, specifically by discussing the event. However, current literature provides no consensus on whether parents and children communicate about PTEs, in what way they might communicate and how this affects the child. Hence the goal of the current study is threefold, to explore: (a) whether parents and children communicate about PTEs, (b) what this communication looks like, and (c) how this affects children’s well-being. These questions are answered by means of a systematic literature review. Articles were eligible for inclusion if it was an empirical study on communication between parents and children about a PTE that the child (under 18 years) had experienced. Initial searches in electronic databases provided 31,233 articles, of which 26 were deemed eligible for inclusion. Results show that most parents and children have discussed PTEs, but that this may depend on cultural background. What the parent–child communication looks like depends on various factors such as, age of the child, tone, and child’s initiation of discussion. Parental post-traumatic stress symptoms seem to negatively impact communication. The results of the impact of communication are less clear-cut, but it seems to have a predominantly positive effect on the child’s well-being, depending on parental sensitivity. Clinicians should be watchful for parental symptoms of post-traumatic stress disorder and can focus on promoting parental sensitivity and responsiveness when discussing PTEs with their child or on creating a joint narrative within families.

A negative experience or situation that threatens a person’s physical and mental well-being can be referred to as a potentially traumatic event (PTE; Turner et al., 1995). Examples of PTEs are natural disasters, (traffic) accidents, or acts of interpersonal violence. Unfortunately, experiencing a PTE is common, even before reaching adulthood. In a normative sample, a prevalence of 14% of exposure to PTE is found in children aged 8 to 12 (Alisic et al., 2008) and even higher (i.e., 50%–90%) in the general adult population of North America (Kilpatrick et al., 2013). The experience of PTE in childhood is associated with increased risk of psychiatric problems in childhood and later in life, such as depression and post-traumatic stress disorder (PTSD; McLaughlin et al., 2010). Luckily, the majority of individuals do not develop subsequent PTSD after experiencing a PTE (Ozer et al., 2003), which may depend on various factors. One of these factors may be communication about the PTE. The current study explored communication about PTEs by performing a systematic review.

## Social Environment

After experiencing a PTE, the social environment plays an important role in an individual’s well-being. Previous research has shown that supportive social networks can buffer negative consequences of stressful events (Charuvastra & Cloitre, 2008). More specifically, the more support individuals experience after a PTE, the less likely they are to develop subsequent post-traumatic stress symptoms (Brewin et al., 2000). Moreover, many studies have confirmed that lack of social support after PTEs is one of the most important predictors of PTSD, as evidenced by various meta-analyses (e.g., Brewin et al., 2000; Zalta et al., 2021). One important source of social support for children is family, more specifically the parent–child relationship (Bowlby, 1969). Indeed, research shows that after PTEs, children who perceived their parents as unavailable experienced increased PTSD symptoms (Scheeringa & Zeanah, 2005). In addition, more parent–adolescent conflict following a natural disaster, predicted more subsequent adolescent PTSD symptoms (Bountress et al., 2020). Thus the parent–child relationship seems to play an important role in well-being after experiencing a PTE.

While the parent–child relationship can be an important buffer after a PTE, evidence suggests that this relationship can also be negatively impacted after experiencing such an event. This is in line with the support deterioration model, which poses that highly stressful events can result in a decrease in perceived support from family (Barrera, 1986). For example, Daniels and Bryan (2021) found that young adults with higher exposure to PTEs, experienced less family cohesion. In addition, studies found that higher PTSD symptoms in adolescents are associated with more parent–child conflict four months later (Bountress et al., 2020) and more parental psychopathology (Forresi et al., 2021). Thus, the general parent–child relationship seems to be impacted by the experience of a PTE, while a supportive parent–child relationship can be beneficial to the child’s well-being after experiencing a PTE.

## Parent–Child Communication

One specific aspect of the parent–child relationship is communication about PTEs, which may play a vital role in their well-being after experiencing a PTE. When the discloser chooses to talk about a PTE they experienced, it is thought to be beneficial for the mental health of both discloser and recipient. Talking helps others to understand the thoughts and feelings of the victim and provide social support (Stein et al., 2017). However, as Ullman (2011) pointed out, how helpful communication is on PTEs can depend on various factors. For example, disclosure of sexual abuse was related to more positive outcomes for the discloser when the disclosure was more detailed and happened immediately after their experience (Ullman, 1996). Moreover, children who disclosed sexual abuse to their parents (vs. disclosure to others) showed fewer depressive and PTSD symptoms (Ullman, 2003). A recent meta-analysis, including different types of PTEs also found that parental avoidance of PTE communication is detrimental for the child’s well-being (Afzal et al., 2023). Taken together, parent–child PTE communication seems to contribute to the processing of the PTE.

However, communication about PTEs is not standard practice within every family. In general, disclosure about PTEs as well as thoughts and feelings about these events seemed to be low in families with adolescents, despite adolescents indicating they had experienced numerous PTEs (Acuna & Kataoka, 2017). The same was found in a study with younger children and their mothers. Although PTEs had been experienced, these were not often discussed (Overbeek et al., 2019). On the other hand, there are also studies that found that most parent–child dyads have talked about PTEs they had experienced (Alisic et al., 2012). Furthermore, Ackil et al. (2003) found that conversations about PTEs between mother and child were longer and more detailed than conversations about non-traumatic events, indicating more elaborate PTE communication. In conclusion, there are contradicting findings in the current literature with regard to what extent parents and their children communicate about PTEs at all.

## Communication Patterns and Impact on Well-Being

Even in parent–child dyads where there is communication about PTEs, the way parents and children communicate with one another may differ between families. For example, Dalgaard et al. (2016) found four different communication styles about PTEs in refugee families, which may also result in different outcomes for the adjustment of the child. In a review, Dalgaard and Montgomery (2015) found that most studies indicated that modulated disclosure (developmentally timed communication carried out in a sensitive matter) of parental PTE was associated with the most positive outcomes for children, while silencing was found to be harmful. So it seems that the way parents and children communicate about parental PTE impacts child adjustment. Moreover, similar findings emerge from studies in which children are the ones who have experienced a PTE. Indeed, Ullman (2003) has found that children showed better psychological adjustment when parents had an accepting attitude toward the PTE and provided socioemotional support (e.g., non-blaming, facilitating PTE talk, and listening). However, talking about PTEs too much can also have detrimental effects. Studies have shown that rumination, talking about PTEs excessively and constantly, was related to poorer adjustment (Stroebe et al., 2006). Additionally, it is not yet clear how children are affected by discussion of a PTE when the event is one shared by both parent and child. To further the understanding on parent–child communication patterns about PTEs and their impact on well-being, a careful review of the literature is needed.

## The Current Study

The goal of this study is to paint an overarching picture of communication patterns between parents and non-adult children about PTEs experienced by either child or both children and parents, as well as understand the impact of this communication on the child’s well-being. Such insight is important because dysfunctional communication patterns within families may hinder recovery and exacerbate symptoms (Eruyar et al., 2020). In this way, the current study could help to identify targets for prevention and intervention for families or individuals within families who experience a PTE. A systematic review is performed, attempting to answer three research questions: (a) Do parents and children talk about the experience of a PTE? (b) How do parents and children communicate about PTEs? and (c) How does parent–child PTE communication affect children’s well-being?

## Method

This study is reported based on the Preferred Reporting Items for Systematic Reviews and Meta-Analyses guidelines (Moher et al., 2009).

### Operationalization

In the current literature, the terms “trauma” and “traumatic event” are often used to describe a negative experience that threatens a person’s well-being. In the strict sense of the definition (American Psychological Association, 2013), such an experience can only be called a “trauma” or a “traumatic event” if the individual develops a stress response after the event in the form of symptoms, hence the use of the word “potentially” in PTE (Krupnik, 2019). However, in the literature the terms “trauma” or “traumatic event” are often used interchangeably with PTE, even when it is not clear whether subsequent PTSD symptoms have occurred. In the current study, the term PTE has been used where the individual articles may use the terms “trauma” and “traumatic event.” To determine whether an article was eligible for inclusion in the current study, it was assessed whether the studied PTE would meet the definition of a PTSD-related event as outlined in the International Classification of Diseases (World Health Organization, 2019): “Exposure to an event or situation (either short- or long-lasting) of a threatening or horrific nature.”

Additionally, to be included, an article needed to report some type of parent–child communication about this PTE. This communication could be measured in any possible way. For example, a parent being interviewed on how they discuss the PTE with their child, asking the child how they communicate with their parent about the PTE, or researchers specifically instructing parents and children to discuss the PTE as part of the study. Studies using general parent–child communication measures (not about the PTE) or communication about the PTE with different people (not parent–child) were not included.

The final goal of the current study was to determine the impact of parent–child PTE communication on child well-being. This is a broad term that roughly encompasses both feeling good and functioning well (Huppert, 2017). PTEs can have various consequences, besides the experience of PTSD symptoms. Therefore, all measures that assessed how a child feels or functions were included as measure of well-being in the current study.

### Eligibility Criteria

The current review aimed to identify empirical articles on parent–child communication about PTEs. Specific inclusion criteria were: Either children or both children and parents experienced a PTE (a), communication on the PTE was measured as informed by parents, children, or both (b), children were under 18 years of age (c), an empirical study was performed, and (d) it was written in English (e). Studies could not be included if the PTE was not the topic of communication (a), if only parents had experienced a PTE (b), communication between other individuals (rather than between parents and children) was measured (c), children were adults, or (d) if the article was a meta-analysis or review (e).

### Search Strategy

A combination of keywords was used to screen the databases MEDLINE, Web of Science, PubMed, PsycINFO, and Embase for potential articles. The search string included the following keywords: (“posttraumatic stressdisorder” OR “post traumatic stress disorder” OR “posttraumatic stress disorder” OR “posttraumatic stress disorder” OR “post-traumatic stress disorder” OR “PTSD” OR “psychotrauma” OR “psycho-trauma” OR “psycho trauma” OR “psychological trauma” OR “life-events” OR “life events” OR “traumatic events” OR “traumatic exposure” OR “PTE” OR “trauma”) AND (“communicat*” OR “talk*” OR “convers*” OR “exchange” OR “discuss*” OR “debate” OR “express” OR “disclos*”) AND (“child*” OR “kid*: OR “adolescen*” OR “parent” OR “mother” OR “mom” OR “father” OR “dad” OR “family” OR “transgenerational” OR “intergenerational”). Various combinations and spellings were used for each keyword and database to ensure all relevant articles would be included. The initial searches were completed in April 2022.

### Analytic Strategy

After removing duplicates, articles were first screened for eligibility. The first phase of screening consisted of screening based on title and abstract. Second, the remaining articles were screened in full text to assess whether all inclusion criteria were met. Any doubts about inclusion were resolved through discussion between authors. When articles were deemed eligible for inclusion, the reference list was screened to identify additional articles that could be included. All included articles were rated on their quality. Since the review included studies with different approaches, the Mixed-Methods Appraisal Tool (Hong et al., 2018) was used. This method allows the appraisal of the quality of qualitative, quantitative, and mixed-methods studies. The appraisal was performed individually by the first author and a research assistant. Any disagreements were resolved through discussion.

## Results

### Search Results

**Figure 1.**
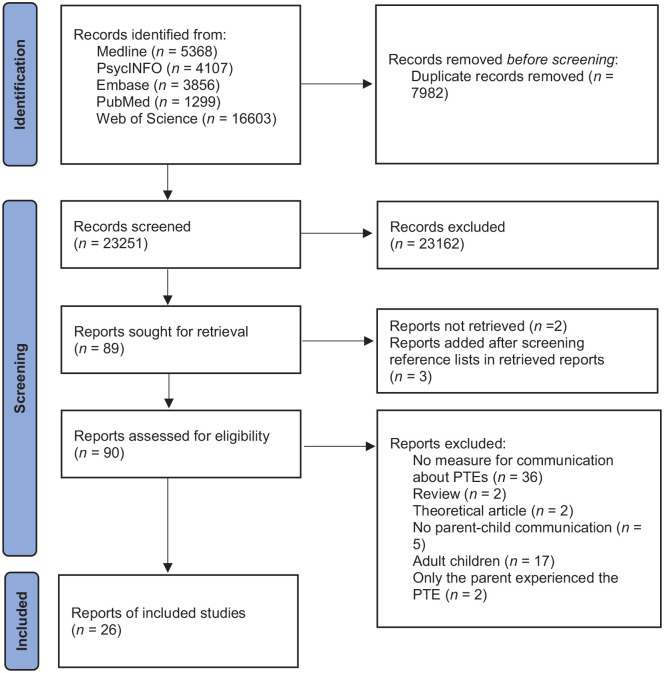
Flow diagram (Page et al., 2021).

The initial search resulted in a total of 31,233 articles. After the removal of duplicates, 23,251 articles were screened. Of the 92 articles that were eligible for full-text assessment, 26 were included in the current study (Figure 1). The risk of bias assessment revealed a satisfactory quality of the included articles (see Table 1), with 14 studies meeting all five criteria. For the remaining studies, satisfaction of one or two criteria was either not met (*n* = 6) or unclear (*n* = 6). The most common risk of bias was the risk of nonresponse bias in quantitative studies.

**Table 1.** Risk of Bias Assessment.

Studies	Category	Methodological Quality Criteria				
		1	2	3	4	5
Abel et al. (2021)	4	Yes	Yes	Yes	Cannot tell	Yes
Ackil et al. (2003)	4	Yes	Yes	Yes	No	Yes
Acuna and Kataoka (2017)	4	Yes	Cannot tell	Yes	Yes	Yes
Alexander et al. (2004)	1	Yes	Yes	Cannot tell	Yes	Yes
Alisic et al. (2012)	1	Yes	Yes	Yes	Yes	Yes
Alisic et al. (2017)	4	Yes	Yes	Yes	No	Yes
Canale et al. (2022)	3	Yes	Yes	Yes	Yes	Yes
Carpenter et al. (2017)	4	Yes	Yes	Yes	No	Yes
Cohodes et al. (2021)	4	Yes	Yes	Yes	Cannot tell	Yes
Dalgaard et al. (2016)	5 (1 + 4)	Yes	Yes	Yes	Yes	No
Dalgaard et al. (2019)	5 (1 + 4)	Yes	Yes	Yes	Yes	Yes
Felix et al. (2020)	4	Yes	Yes	Yes	Cannot tell	Yes
Field et al. (2014)	4	Yes	Yes	Yes	No	Yes
Garfin et al. (2014)	4	Yes	Yes	Yes	Yes	Yes
Gil-Rivas et al. (2007)	4	Yes	Yes	Yes	No	Yes
Hafstad et al. (2012)	1	Yes	Yes	Yes	Yes	Yes
Hendrickson et al. (2020)	4	Yes	Yes	Yes	Yes	Yes
Lindgaard et al. (2009)	1	Yes	Yes	Yes	Yes	Yes
McGuire et al. (2019)	5 (1 + 4)	Yes	Yes	Yes	Yes	Yes
Montgomery (2004)	1	Yes	Yes	Yes	Yes	Yes
Murphy et al. (2016)	4	Yes	Yes	Yes	Yes	Yes
Murphy et al. (2021)	4	Yes	Yes	Yes	Yes	Yes
Overbeek et al. (2019)	4	Cannot tell	Yes	Yes	Yes	Yes
Williamson et al. (2016)	1	Yes	Yes	Yes	Yes	Yes
Williamson et al. (2017)	1	Yes	Yes	Yes	Yes	Yes
Williamson et al. (2019)	1	Yes	Yes	Yes	Yes	Yes

### Participants

The 26 included studies reported on a total of 2,711 children (45% female) and 2,803 parents (80% mothers). Children were on average between 2 and 17 years old (*M* = 11.74, *SD* = 2.57) and parents were on average 40.94 years old (*SD* = 7.34).^1^ Type of PTE varied between studies, including experiencing natural disasters (*n* = 7), war (*n* = 1), being a refugee (*n* = 2), terrorism (*n* = 2), accident with injury (*n* = 3), death/illness (*n* = 3), (sexual) violence (*n* = 1), or unspecified/various PTEs (*n* = 7). In most studies, the parent–child dyads had experienced the PTE together (*n* = 17). In the remaining studies, only the child (*n* = 9) had experienced the event. Most of the studies adopted a quantitative approach (*n* = 15); eight adopted a qualitative approach, and the remaining three adopted a mixed-methods approach. See Table 2 for more details on the included studies.

**Table 2.** Results: The Included Studies.

Article	Type of Study	Country	Type of PTE	Well-being Measure	Participant	*N*	Age *M* (*SD*)	Female (%)
Abel et al. (2021)	Quantitative	USA	Tornado	PTSD symptoms	Child	49	9.4 (1.38)	49.0
					Parent	49	35.8 (—)	100
Ackil et al. (2003)	Quantitative	USA	Tornado	—	Child	29	6.7 (—)	37.9
					Parent	22	—	100
Acuna and Kataoka (2017)	Quantitative	USA	Various	PTSD symptoms	Child	98	13.2 (1.17)	44.9
					Parent		—	—
Alexander et al. (2004)	Qualitative	USA	Adoption	Psychiatric symptoms and self-esteem	Child	25	13.0 (—)	28.0
					Parent	35	49.0 (—)	68.6
Alisic et al. (2012)	Qualitative	The Netherlands	Various	Parent interview	Child	25	10.7 (1.04)	40.0
					Parent	33	41.4 (5.80)	73.0
Alisic et al. (2017)	Quantitative	Australia	Injury	Psychiatric symptoms	Child	71	10.4 (3.60)	40.8
					Parent	132	—	52.3
Canale et al. (2022)	Quantitative	USA	Various	Psychiatric symptoms and PTSD symptoms	Child	71	12.3 (2.70)	69.0
					Parent	71	43.0 (12.00)	90.0
Carpenter et al. (2017)	Quantitative	USA	Boston marathon bombing	PTSD symptoms	Child	460	11.8 (3.80)	92.2
					Parent	460	35.4 (4.50)	—
Cohodes et al. (2021)	Quantitative	USA	COVID-19	Psychiatric symptoms	Child	200	8.8 (4.78)	52.5
					Parent	200	38.3 (7.32)	54.0
Dalgaard et al. (2016)	Mixed methods	Denmark	Refugee	Psychiatric symptoms and PTSD symptoms	Child	30	6.8 (1.55)	46.7
					Parent	56	—	—
Dalgaard et al. (2019)	Mixed methods	Gaza strip	War	Psychiatric symptoms and PTSD symptoms	Child	170	11.2 (0.80)	49.4
					Parent	340	37.4 (7.40)	50.0
Felix et al. (2020)	Quantitative	USA	Flood	Depressive symptoms	Child	485	13.8 (2.56)	47.2
					Parent	485	—	69.0
Field et al. (2014)	Quantitative	Cambodia	Death of father	Self-esteem and adaptive grief	Child	48	15.7 (1.62)	54.2
					Parent	48	—	100
Garfin et al. (2014)	Quantitative	Chile	Earthquake	PTSD symptoms	Child	118	7.6 (0.65)	43.2
					Parent	118	—	—
Gil-Rivas et al. (2007)	Quantitative	USA	9/11	PTSD symptoms	Child	104	15.2 (1.30)	47.1
					Parent	104	45.0 (7.90)	50.0
Hafstad et al. (2012)	Qualitative	Norway	Tsunami	—	Child	80	12.2 (3.50)	50.0
					Parent	51	43.1 (5.20)	78.4
Hendrickson et al. (2020)	Quantitative	USA	Tornado	PTSD symptoms	Child	122	14.5 (1.30)	34.0
					Parent	122	—	100
Lindgaard et al. (2009)	Qualitative	Norway	Tsunami	Parent interview	Child	130	13.0 (—)	—
					Parent	84	43.8 (—)	73.8
McGuire et al. (2019)	Mixed methods	United Kingdom	Injury	PTSD symptoms	Child	20	9.0 (2.30)	30.0
					Parent	20	38.8 (7.70)	95.0
Montgomery (2004)	Qualitative	Denmark	Refugee	—	Child	8	10.6 (—)	50.0
					Parent	6	—	50.0
Murphy et al. (2021)	Quantitative	USA	Cancer	—	Child	67	10.0 (3.63)	46.0
					Parent	67	38.2 (6.84)	100
Murphy et al. (2016)	Quantitative	USA	Cancer	PTSD symptoms	Child	41	13.3 (2.33)	54.0
					Parent	41	41. 8 (7.83)	100
Overbeek et al. (2019)	Quantitative	The Netherlands and Israel	Violence	—	Child	213	9.7 (2.60)	48.8
					Parent	213	40.8 (5.45)	100
Williamson et al. (2017)	Qualitative	South Africa	Various	—	Child	20	11.5 (3.02)	55.0
					Parent	20	41.3 (8.02)	100
Williamson et al. (2016)	Qualitative	United Kingdom	Injury	PTSD symptoms	Child	20	10.4 (3.20)	40.0
					Parent	20	41.6 (6.1)	75.0
Williamson et al. (2019)	Qualitative	United Kingdom	Various	PTSD symptoms	Child	7	11.4 (2.30)	29.0
					Parent	6	41.3 (7.80)	71.0

*Note.* PTE = potentially traumatic event; PTSD = post-traumatic stress disorder.

### The Prevalence of Communication

First, it was explored whether parents and children communicated about the PTE at all. In 16 of the 26 included studies, a very large majority (> 80%) or the entire sample indicated that they had communicated about PTEs (Abel et al., 2021; Ackil et al., 2003; Alexander et al., 2004; Alisic et al., 2012, 2017; Canale et al., 2022; Cohodes et al., 2021; Dalgaard et al., 2016; Hafstad et al., 2012; Hendrickson et al., 2020; Lindgaard et al., 2009; McGuire et al., 2019; Murphy et al., 2016; Murphy et al., 2021; Williamson et al., 2016, 2019). In four of the 26 studies, there were mixed results with about half of the parents or children indicating no communication about the PTE, while the other half did report previous PTE communication (Dalgaard et al., 2019; Field et al., 2014; Gil-Rivas et al., 2007; Williamson et al., 2017). These findings are based on interviews with parents or children in which they were asked “How much/How often do you communicate with your child/parent about this event?”. There were also a few studies indicating the PTE was rarely discussed (Acuna & Kataoka, 2017; Montgomery, 2004; Overbeek et al., 2019) or that did not specify the amount of communication (Carpenter et al., 2017; Felix et al., 2020; Garfin et al., 2014).

### Patterns of Communication

Of the 26 included studies, 17 also reported more details on what PTE communication looks like. Several studies reported that parents were actively trying to encourage their child to discuss what happened at their own pace and in a safe space (Alisic et al., 2012; Carpenter et al., 2017; Hafstad et al., 2012; McGuire et al., 2019; Williamson et al., 2016, 2019). They did this by asking the child how they feel, encouraging them to share their experience, and providing the opportunity for the child to ask questions. In some studies, parents also employed a strategy of reassurance, where they would tell their child that they are now safe as well as to normalize any feelings the child may have (Carpenter et al., 2017; Williamson et al., 2017).

The pattern of communication seemed to depend on various factors, such as the age of the child. In one study, the majority of the parents indicated they discussed the PTEs in age appropriate ways while trying to take their child’s perspective (Dalgaard et al., 2016). However, Canale et al. (2022) found that caregivers were more likely to express blame or criticism of the PTE toward older children. Furthermore, the pattern of communication also seemed to depend on the parent. Fathers were found to talk very little or not at all about a shared PTE with their child (Montgomery, 2004). Moreover, children were more likely to discuss the PTE and perceive it as helpful when communicating with their mother compared to their father (Alisic et al., 2017) or to other relatives (Field et al., 2014).

In addition, results showed contrasting findings on the tone of the communication. On one hand, Ackil et al. (2003) found that mothers were more likely to mention negative emotions (i.e., sadness, fear, anger) in conversations about the PTE as compared to conversations about non-traumatic events. In contrast, Alisic et al. (2017) found that the conversation tone was more positive when mothers were talking to their child about PTEs than in other conversations. Yet another study found that the mention of positive and negative emotions was correlated. In a conversation about the PTE, increased mentioning of positive emotions was associated with increased mentioning of negative emotions (Abel et al., 2021). Dalgaard et al. (2019) also found both positive (future prospects) and negative (violent details) communication between parents and children. So the tone of parent–child PTE communication can have both positive and negative elements. Some studies also provided insight into what may predict conversation tone. Higher maternal PTSD was associated with more harsh and withdrawn communication toward their child (Murphy et al., 2016) and less expression of positive emotions during the conversation (Murphy et al., 2021). Therefore, it seems that parent’s well-being may also impact the pattern of communication.

Finally, some studies also reported reasons for not communicating about PTEs. The most commonly listed reason for not discussing the PTE was parents not wanting to upset their child (McGuire et al., 2019; Williamson et al., 2016, 2017, 2019) or children not wanting to upset the parent (Field et al., 2014). McGuire et al. (2019) found that most parents in their study felt it was best to let the child initiate discussion of the PTE. Other studies however also found that some parents actively avoided discussion of the event (Williamson et al., 2016, 2017) and even encouraged other family members to do so as well (Williamson et al., 2019). Thus, it seems that a few parents adopted an avoidance approach, even when children initiated the PTE communication. Some parents also indicated they avoided discussing the PTE because they felt they would be unable to adequately support their child (Hafstad et al., 2012; Williamson et al., 2019). In one study, the majority of the parents indicated they had discussed it once, but had not talked about it anymore since, because parents expected the child to bring it up if they wanted to talk about it further (Alexander et al., 2004). Finally, it was found that PTE communication was more likely to occur when adolescents perceived their parents to be more open and receptive (Acuna & Kataoka, 2017).

### Impact of Communication

The final aim of this study was to explore how children are affected by the patterns of parent–child communication about PTEs. Of the 26 included studies, 20 have explored how the parent–child PTE communication could affect children. The most common way to measure well-being was by assessing PTSD symptoms (*n* = 13). Other measures tapped into general psychiatric symptoms (*n* = 6), adaptive grief (*n* = 1), depressive symptoms (*n* = 1), and self-esteem (*n* = 2). In some studies parents reported on their child’s well-being in an interview. A few studies included more than one measure of well-being. For an overview of the well-being measures in each study, see Table 2.

Overall, it seemed that discussing PTEs in a positive way, such as encouraging the child to express thoughts and feelings and helping them navigate this, was thought to beneficial to children’s well-being (Alisic et al., 2012, 2017; Carpenter et al., 2017; Dalgaard et al., 2019; Lindgaard et al., 2009; McGuire et al., 2019), whereas not discussing the PTE was harmful to children’s recovery from the event (Carpenter et al., 2017; Felix et al., 2020; Garfin et al., 2014; Gil-Rivas et al., 2007). However, what is considered helpful communication differed between studies. Some studies stated that more communication predicted better adjustment. Alisic et al. (2017) found that more conversations about the PTE with parents were related to fewer peer and conduct problems for the child at a later moment, regardless of the quality of those conversations. Parents also stated that they believed discussing the event would help support their child process it (McGuire et al., 2019). In a different sample, parents also identified talking about the event as an important strategy to facilitate recovery of their child (Alisic et al., 2012). Hence, these studies suggest that more parent–child communication about the PTE predicts higher well-being for the child.

However, other studies did not find such a relationship between parent–child communication and child adjustment. Acuna and Kataoka (2017) found that actual discussion of events between parents and children was not related to PTSD symptoms, but general parent–child communication problems were. Moreover, parent–child discussion of PTEs did not buffer the stress that children experience (Cohodes et al., 2021). In addition, one study indicated that positive PTE communication did not affect child adjustment, but negative PTE communication—such as expressing criticism—did (Canale et al., 2022). Finally, some studies even suggest that certain types of communication may be harmful. For example, Dalgaard et al. (2016) found that parents adopting an “unfiltered speech” approach (openly discussing the PTE without realizing their child is listening) are more likely to have insecurely attached children. Moreover, higher scores of parent–child co-rumination are related to higher PTSD symptoms in children (Felix et al., 2020). In one study, parents also expressed concerns about discussing the event too often, fearing it would cause a strain on their relationship with their child (Alexander et al., 2004). Taken together, these studies suggest that there may be an optimal amount or type of PTE communication.

This becomes apparent in various studies that have explored perceived helpfulness of communication. Field et al. (2014) found that adolescents scored higher on self-esteem and adaptive grief (as opposed to traumatic/complicated grief), when they perceive PTE communication with their mother as more helpful. In addition, perceiving discussion of events with parents as unhelpful was associated with more PTSD symptoms in adolescents (Gil-Rivas et al., 2007). Thus, communication that is perceived as helpful is especially beneficial for children’s well-being. One factor that might play a role in perceived helpfulness of parent–child communication could be cohesiveness of the joint narrative of the event. Lindgaard et al. (2009) found that families who constructed a narrative of the event together experienced more cohesiveness. Disagreements about the events appeared to be a source of conflict. Montgomery (2004) also suggests that talking about these events could create more cohesion and clarity for children.

Another factor that may play a role in the perceived helpfulness in parent–child PTE communication, may be parental responsiveness toward the experience of the child in such a conversation. Abel et al. (2021) found that PTSD symptoms were higher in children when their mother did not acknowledge their child’s emotion during a PTE conversation. Regardless of whether children talked in a positive or negative way about the event, the acknowledgment of those emotions from their mother is key for their adjustment. A similar notion was found by Murphy et al. (2016), who concluded that higher maternal validation of their children’s story when discussing the PTE predicted fewer PTSD symptoms in children. Hendrickson et al. (2020) also found that children experienced more PTSD symptoms, when caregivers centered conversations about PTEs more around themselves, instead of their children’s experiences. In addition, when caregivers expressed blame or criticism toward the child when discussing the event, the child showed poorer adjustment (i.e., more negative emotions and behavior; Canale et al., 2022). Hence, parental responsiveness in PTE communication is perceived as more helpful and plays a role in children’s adjustment.

## Discussion

The goal of the current study was to create an overview of communication patterns between parents and their children about PTEs that either children or both children and parents had experienced. Understanding PTE communication is deemed important as it provides insight into factors that hinder or facilitate recovery from the PTE. The results showed that the majority of parents and children have discussed the PTE. The way parents and children communicate about PTEs varied depending on children’s age, the communication tone, and children’s role in initiating discussion. Findings about the impact of communication were less clear-cut. Parent–child PTE communication seemed to have a predominantly positive impact on children’s well-being, though this depended on parental responsiveness, perceived helpfulness, and cohesion of the joint narrative. The results for each research question are discussed in more detail below.

### Main Study Results

The first aim of the current study was to explore how many parents and children engage in discussion of PTEs. The results showed that parents and children generally do discuss PTEs. However, there were a few studies that found only half of the sample or barely any parents and children had discussed the PTE together. When zooming in on these studies, it becomes apparent that samples with less parent–child PTE communication more often included individuals and families from non-westernized countries. These studies took place in the Gaza Strip (Dalgaard et al., 2019), Cambodia (Field et al., 2014), Israel (Overbeek et al., 2019), South Africa (Williamson et al., 2017), and in Denmark with Middle Eastern refugees (Montgomery, 2004). There was one such study which took place in the United States, but their sample only included a small percentage of White/Caucasian individuals (18%; Acuna & Kataoka, 2017). In contrast, studies that did find the majority of their sample had communicated about PTEs took place in Western countries such as the United States, the United Kingdom, the Netherlands, or Scandinavian countries and included more homogenous samples. Previous research has found that the expression of one’s inner state and feelings is more encouraged in Western cultures, while this may be different in other cultures. For example, in Eastern cultures individuals are more likely to modify their emotional expression to not influence others (Lim, 2016). Reservation in expressing one’s inner thoughts is often promoted in Eastern cultures (Tsai et al., 2007). Furthermore, discussing a PTE can be viewed as poor adjustment in some non-Western cultures (Williamson et al., 2017), possibly explaining why families from non-Western cultures are less likely to discuss PTEs. Hence, the differences in prevalence of parent–child communication that were found in the current study could be due to cultural differences.

The second research question focused on the patterns of PTE communication between parents and children. The tactic that most parents seemed to employ was that of actively encouraging conversations about the PTE, for example, by asking their child how they feel about the event. However, there were also a few studies that included parents who actively avoided discussing the PTE with their child. When looking more closely at these studies, it is striking that the groups of parents who actively promoted discussion and those who actively avoided it were found within the same studies (Williamson et al., 2016, 2017, 2019; McGuire et al., 2019). These researchers posed that this could be due to parental symptoms of PTSD (Williamson et al., 2016). For example, one longitudinal study showed that parents experienced persistent PTSD symptoms for years after they discovered their children were sexually abused (Tsang et al., 2021). When parental distress is high, avoidance could be part of the parent’s own coping strategies, because it is too painful for them to discuss the PTE (Hiller et al., 2016). Their distress may also leave parents less sensitive toward their child’s distress and their potential need to discuss the PTEs, because parents are preoccupied with their own symptoms (Meiser-Stedman et al., 2007). Therefore, parental symptoms of PTSD may play a role in the employment of avoidance strategies. Additionally, parental PTSD symptoms also play a role in the conversation tone. Mothers with higher PTSD symptoms are more likely to show harsher communication toward their child (Murphy et al., 2016) and less likely to express positive emotions (Murphy et al., 2021) when discussing PTEs. In conclusion, parental symptoms of PTSD seem to play an important role in the tone of the conversation as well as choosing communication strategies (either of avoidance or active encouragement).

Finally, the aim was to explore how children are impacted by communicating with their parents about PTEs. Generally, parents in most studies agreed that communication about the PTE was beneficial for the child’s well-being and recovery (e.g., Alisic et al., 2012) or at least that refraining from such conversation was harmful, resulting in higher PTSD symptoms in the child (Carpenter et al., 2017). However, some studies suggested that certain types of communication could be harmful. Specifically, parental sensitivity and responsiveness in PTE conversations with children were predictive of the impact of communication. Indeed, the results suggested that children showed worse adjustment when parental sensitivity and responsiveness were low (Murphy et al., 2016; Hendrickson et al., 2020; Abel et al., 2021). Hence, results suggest that it is especially important for the parent to be sensitive toward their child’s thoughts and emotions during conversations about the PTE. Sensitivity can increase responsivity through praising, empathizing, and confirming children’s expressions. Increasing parental sensitivity and responsiveness may then mold the joint narrative of the event and increase familial cohesiveness (Lindgaard et al., 2009). In conclusion, parental sensitivity and responsiveness seems to play an important role in the impact of parent–child PTE communication.

### Limitations

Although the results of the current study bring a new perspective on parent–child PTE communication and its impact, they should be interpreted within the context of few limitations. First, although the overall quality of the included studies was good, some were at risk for bias. The most prevalent bias was the risk of nonresponse bias in quantitative studies (Alisic et al., 2017; Carpenter et al., 2017; Field et al., 2014; Gil-Rivas et al., 2007). In these cases it was unclear whether participants of the study differed in any way from those who did not participate (e.g., refusals; Hong et al., 2018). This could lead to a biased sample, where those who chose to participate may be more open to discuss their PTE in general, while refusal could also be a strategy of avoidance. While the risk of nonresponse bias was not present in the majority of the studies, it is important to keep in mind this potential bias in some of the samples.

Second, the patterns of communication could not be explored as a function of age. Parents are known to communicate with their children in vastly different ways, depending on the age of the child (Vangelisti, 2012), and this may also be the case with communication about PTEs. For example, Dalgaard et al. (2016) found that most parents try to discuss PTEs in age appropriate ways. However, because the age variations of the included participants within and between the included studies were so large, no other communication patterns dependent on the age of the child could be explored.

Third, the current study was unable to assess whether parent–child communication varied due to type of PTE. It is imaginable that in cases of interpersonal events, such as sexual abuse, children may find it more difficult to discuss the event due to a difficulty to trust others. Moreover, parents may also find it more difficult to discuss such events due to feelings of shame and blame. Unfortunately, there were too many different PTEs and too few within each category to compare them and make useful conclusions about this.

Finally, the current study only included samples where children were younger than 18 years of age. To also include samples on parent–child communication with adult children is beyond the scope of the current study but may also lead to additional insights.

### Implications for Practice and Future Research

Despite these limitations, the results have important implications (see Table 3). First of all, parental sensitivity and responsiveness during conversations on PTEs was found to be crucial for the child’s well-being. With this knowledge, clinicians can be sure to assess and target these factors when working with children who have experienced PTEs. Previous studies have shown that abilities such as showing sensitivity and responsiveness toward the child’s experience (by affirmation, empathy, and praise) are something that parents can learn (Slade, 2007). However, even when working on parental sensitivity and responsiveness, it is important to be mindful that these skills may not always transfer to communication about PTEs. This type of communication is not part of standard training programs for parents. Moreover, it is not possible to say what is the single best practice of PTE communication based on the current literature. Therefore, it is important for clinicians to coordinate with parents about which type of PTE communication is most sensitively attuned to the child’s needs and would contribute to their child’s well-being. The current review offers a starting point for clinicians to know what elements they could address in parent–child PTE communication. In addition, clinicians may want to focus on improving the joint narrative of the PTE as this is beneficial for both the family as a whole and the individual family members (Lindgaard et al., 2009). Moreover, clinicians should also be aware of cultural differences when seeing families who have experienced a PTE as they may have different views on PTE communication. Finally, it is important to explore whether parents may be experiencing PTSD symptoms, as this seems to impact communication in a negative way and can even occur when only the child has experienced the event. Clinicians should be aware that parents may experience PTSD symptoms that require separate treatment, because of their impact on the child’s well-being.

**Table 3.** Implications for Practice, Policy, and Research.

Clinicians should focus on improving parental sensitivity toward their children in PTE conversations.
Clinicians can focus on improving the joint narrative of the PTE within families.
Both researchers and clinicians should take into account cultural differences in treatment or research of families who have experienced PTEs.
Policymakers can encourage the focus on parental post-traumatic stress symptoms in treatment and encourage research on the communication strategies associated with parental symptoms of PTSD.
Research can explore PTE communication between parents and adult children.

*Note.* PTE = potentially traumatic event; PTSD = post-traumatic stress disorder.

In addition to the practical implications, the current study also creates new avenues to be further explored in future research. First of all, future research should consider the cultural background of individuals engaging in PTE communication. The prevalence of PTE communication may differ as a function of culture, depending on their norms for communicating one’s inner thoughts and feelings (Lim, 2016). Future studies should explore whether this is indeed the case. Second, future studies should explore why parents choose certain strategies for (not) discussing the PTE with their child. While Williamson et al. (2016) suggest that this may be due to parental symptoms of PTSD, this has not yet been confirmed in other studies. Therefore, research should focus on whether parental symptoms of PTSD can predict avoidance or active encouragement strategies in parent–child PTE communication. Finally, future research should focus on parent–child communication with adult children. The current study focused on children younger than 18 years, but communication may be different when the children are older and less dependent on their parent. There have been studies on parent–child communication in adult children, mainly in offspring of Holocaust survivors (e.g., Braga et al., 2012). However, there are fewer studies on communication when the adult child is the one who has experienced the PTE. Hence, future studies could explore how adult children communicate with their parent about PTEs and how this may be different from communication with younger children.

## Conclusion

In conclusion, most parents and children do communicate about a PTE, but this may depend on cultural background. In addition, the pattern of communication may differ as a function of parental symptoms of PTSD. Finally, parent–child PTE communication seems to be beneficial for children’s well-being in most cases, although this may depend on parental sensitivity and responsiveness. These results have important implications for clinical practice, as well as for future research.

### Diversity in Included Studies

This systematic review included limited diversity across the different studies regarding the geographical location. Most studies took place in Western countries, most often in the United States. There was variety in the participants who were included. Children were of all ages varying from primary school age to late adolescence. This also assured some variety in age of the parents, although most were between 30 and 40 years old. As for gender of the child, there was a 50/50 division overall with some studies including fewer girls and some studies including more girls. As for parents, there was less diversity as the mother was most often the participating parent. In addition, there is ample diversity in type of study, type of PTE, and sample size.

## Notes

1. These statistics are not based on all 26 articles. Not all articles provided the descriptive statistics mentioned here. Corresponding authors were contacted, but this did not yield the desired result. Hence, the descriptive statistics reported in the current study were calculated based on the articles in which this statistics was reported. This becomes apparent in Table 2.
